# Sex and kidney ACE2 expression in primary focal segmental glomerulosclerosis: A NEPTUNE study

**DOI:** 10.1371/journal.pone.0252758

**Published:** 2021-06-07

**Authors:** Nicholas A. Maksimowski, James W. Scholey, Vanessa R. Williams, Nephrotic Syndrome Study Network (NEPTUNE)

**Affiliations:** 1 Institute of Medical Science, University of Toronto, Toronto, Ontario, Canada; 2 Department of Physiology, University of Toronto, Toronto, Ontario, Canada; 3 Division of Nephrology, University Health Network, Toronto, Ontario, Canada; 4 Toronto General Hospital Research Institute, University Health Network, Toronto, Ontario Canada; University Medical Center Utrecht, NETHERLANDS

## Abstract

**Background:**

Angiotensin-converting enzyme 2 (ACE2) has been implicated in the pathogenesis of experimental kidney disease. ACE2 is on the X chromosome, and in mice, deletion of ACE2 leads to the development of focal segmental glomerulosclerosis (FSGS). The relationship between sex and renal ACE2 expression in humans with kidney disease is a gap in current knowledge.

**Methods:**

We studied renal tubulointerstitial microarray data and clinical variables from subjects with FSGS enrolled in the Nephrotic Syndrome Study Network (NEPTUNE) study. We compared relationships between ACE2 expression and age, estimated glomerular filtration rate (eGFR), urinary albumin to creatinine ratio (UACR), interstitial fibrosis, tubular atrophy, and genes implicated in inflammation and fibrosis in male and female subjects.

**Results:**

ACE2 mRNA expression was lower in the tubulointerstitium of males compared to females (*P* = 0.0026). Multiple linear regression analysis showed that ACE2 expression was related to sex and eGFR but not to age or treatment with renin angiotensin system blockade. ACE2 expression is also related to interstitial fibrosis, and tubular atrophy, in males but not in females. Genes involved in inflammation (*CCL2* and *TNF*) correlated with ACE2 expression in males (*TNF*: *r* = -0.65, *P* < 0.0001; *CCL2*: *r* = -0.60, *P* < 0.0001) but not in females. TGFB1, a gene implicated in fibrosis correlated with ACE2 in both sexes.

**Conclusions:**

Sex is an important determinant of ACE2 expression in the tubulointerstitium of the kidney in FSGS. Sex also influences the relationships between ACE2, kidney fibrosis, and expression of genes involved in kidney inflammation.

## Introduction

The renin angiotensin system (RAS) plays a key role in the progression of chronic kidney disease (CKD), and blockade of the RAS is a centerpiece of the current clinical approach to the treatment, especially in CKD associated with proteinuria. Our understanding of the RAS and angiotensin peptide processing is evolving. For example, angiotensin-converting enzyme (ACE) 2, expressed in the kidney mainly proximal tubules and podocytes [1–4], is not targeted by ACE inhibition. ACE2 metabolizes angiotensin (Ang) II to Ang-(1–7), whereas ACE metabolizes Ang I to Ang II [5, 6].

ACE2 plays a role in the pathogenesis of experimental kidney disease. Deletion of the gene for ACE2 leads to the development of proteinuria and focal segmental glomerulosclerosis (FSGS) in mice [7] and exacerbates diabetic kidney injury [8]. Treatment with recombinant ACE2 attenuates diabetic nephropathy [9] and kidney injury in mice with experimental Alport syndrome, a model of progressive proteinuria, kidney inflammation and fibrosis [10]. Interestingly, ACE2 is the cellular receptor for SARS-CoV-2, and this finding has renewed interest in ACE2 expression because it may be a determinant of viral localization and organ injury [11, 12]. There are relatively few studies characterizing ACE2 in human kidneys [2, 13–17].

Sex is an important determinant of kidney outcomes [18–20] and tubulointerstitial injury correlates inversely with glomerular filtration rate (GFR) in primary glomerular diseases like FSGS [21, 22]. We have recently reported that kidney ACE2 expression is lower in males than females in a diverse group of subjects with CKD [23]. Here, we focus on tubulointerstitial expression of ACE2 in female and male subjects with FSGS from the Nephrotic Syndrome Study Network (NEPTUNE) consortium. We compared men and women across a broad set of clinical and laboratory variables including kidney pathology (age, eGFR, UACR, interstitial fibrosis, and tubular atrophy).

## Materials and methods

### Data collection and study cohort

Percutaneous kidney biopsies were obtained from patients after informed consent and with approval of the local ethics committees at each of the participating kidney centers. Written consent and assent were obtained. This covers all aspects of the study including clinical data, biospecimens and any derivatives. Clinical and gene expression information from patients are accessible in a non-identifiable manner. University of Michigan institutional review board in the Department of Medicine (UMich IRBMED) is the institutional review board of record [24].

Biopsies from 111 subjects (45 females and 66 males) with FSGS were microdissected into glomerular and tubulointerstitial components (Tables 1 and 2). Renal biopsy tissue was manually micro-dissected to separate the tubulointerstitial compartment from the glomerular compartment. Total RNA was isolated, reverse transcribed, linearly amplified and hybridized on an Affymetrix 2.1 ST platform as described previously [25, 26]. Gene expression was normalized, quantified, and annotated at the Entrez Gene level.

**Table 1:** Clinical characteristics of subjects with focal segmental glomerulosclerosis.

	All	Male	Female	*P* value
	(*n* = 111)	(*n* = 66)	(*n* = 45)	(Male vs. Female)
**Age at baseline visit**	32.62 ± 20.62	34.92 ± 20.40	29.24 ± 20.71	0.1552
**BMI (kg/m**^**2**^**)**	27.39 ± 7.57	27.94 ± 7.09	26.59 ± 8.24	0.3662
**Sitting Systolic BP (mmHg)**	122.70 ± 16.41	125.80 ± 16.29	118.00 ± 15.63	0.0127
**Sitting Diastolic BP (mmHg)**	74.96 ± 12.76	76.21 ± 12.74	73.13 ± 12.72	0.2137
**Hematocrit (%)**	38.83 ± 5.77	40.42 ± 5.50	36.53 ± 5.43	0.0005
**eGFR (ml/min/1.73 m**^**2**^**)**	72.93 ± 34.06	71.98 ± 33.15	74.36 ± 35.71	0.7212
**Centrally measured timed urine protein (mg/dl)**	199.3 ± 252.8	225.3 ± 271.5	151.0 ± 209.4	0.2038
**Centrally measured timed urine creatinine (mg/dl)**	69.07 ± 48.16	78.65 ± 53.17	51.23 ± 30.54	0.0125
**Centrally measured timed urine albumin (mg/dl)**	1492 ± 1851	1651 ± 1928	1204 ± 1698	0.3081
**Centrally measured timed UPCR**	3.04 ± 4.27	2.90 ± 3.01	3.30 ± 6.00	0.6828
**Centrally measured timed UACR**	2252 ± 2986	2112 ± 2411	2507 ± 3859	0.5770
**Interstitial fibrosis (%)**	20.98 ± 24.33	21.51 ± 25.23	20.18 ± 23.21	0.7964
**Tubular atrophy (%)**	19.44 ± 24.26	19.72 ± 25.24	19.03 ± 23.04	0.8924
**RAASblock (Yes/No)**	79/28	46/17	33/11	

BMI, body mass index; BP, blood pressure; eGFR, estimated glomerular filtration rate; UACR, urine albumin to creatinine ratio; UPCR, urine protein to creatinine ratio; RAASblock, renin-angiotensin-aldosterone system blockers. The Schwartz formula was used to calculate eGFR for subjects less than 18 years of age. The CKD-EPI formula was used to calculate eGFR in adults. Values are presented as the mean ± SD and *P* values were determined by Student’s *t* tests.

**Table 2:** Number of subjects used in each analysis.

	Male	Female	Total
**Age**	62	44	106
**eGFR**	62	43	105
**Sex**	62	44	106
**RAAS Block**	62	44	106
**UPCR**	50	27	77
**Sitting Systolic BP**	62	44	106
**Sitting Diastolic BP**	62	44	106
**IF**	53	37	90
**TA**	53	37	90
**TNF**	53	37	90
**CCL2**	62	44	106
**TGFB**	62	44	106
**COL1A1**	62	44	106
**ACTA2**	62	44	106

ACTA2, actin alpha 2, smooth muscle; CCL2, monocyte chemoattractant protein 1; COL1A1, collagen, type I, alpha 1; eGFR, estimated glomerular filtration rate; IF, interstitial fibrosis; TA, tubular atrophy; TGFB, transforming growth factor beta; TNF, tumor necrosis factor; UPCR, urine protein to creatinine ratio.

Visual assessment was performed according to the Nephrotic Syndrome Study Network Digital Pathology Scoring System (NDPSS), on de-identified whole slide images of renal biopsies according to the NEPTUNE digital pathology protocol (NDPP) [24]. Visual quantitative assessment of IF and TA was reported as 0–100%. Pathological assessment of IF and TA was performed according.

### Statistical analysis

Analyses were performed using GraphPad Prism 7 (GraphPad Software, San Diego, CA). Data are presented as the mean ± SD, unless otherwise stated. Statistical significance was defined as a *P* value of less than 0.05 for the Spearman correlation coefficient analysis and for the multiple regression analysis. Tubulointerstitial median-centered log2 mRNA expression of ACE2 from renal biopsy samples from subjects with FSGS were compared in male and female subgroups. ACE2 expression levels were correlated against age, estimated GFR (eGFR), urine protein to creatinine ratio (UPCR), interstitial fibrosis percentage, and tubular atrophy percentage.

ACE2 expression was also correlated against expression of tumor necrosis factor alpha (TNF), monocyte chemoattractant protein 1 (MCP-1 or CCL2), transforming growth factor beta 1 (TGFB1), collagen, type I, alpha 1 (COL1A1), and actin alpha 2, smooth muscle (ACTA2 or α-SMA) in subjects with FSGS. For comparisons between two groups, two-tailed *P* values were determined by χ^2^ tests for categorical variables and unpaired Student’s *t* tests for continuous variables. Pearson’s correlation coefficient (*r*) with two-tailed *P* values were calculated. Linear regression was used to generate the line of best fit with 95% confidence intervals.

## Results

### Patient characteristics

There were 111 subjects in the FSGS cohort: 66 males and 45 females (Table 1). Although there were missing values for clinical and laboratory parameters (Table 2), we did not input missing values for our analyses. The average age of the group was 32.6 ± 20.6 years with a mean eGFR of 72.9 ± 34.1 ml/min/1.73 m^2^. There was no difference in age or BMI between the male and female subjects. Mean systolic blood pressure and hematocrit values were higher in male subjects compared to female subjects. In terms of kidney function, mean values for eGFR, UPCR, and urine albumin to creatinine ratios (UACR) were similar in males and females. Morphometric measures of kidney interstitial fibrosis and tubular atrophy were also similar (Table 1).

### Correlation of ACE2 mRNA expression with clinical variables in males and females with FSGS

**Fig 1:**
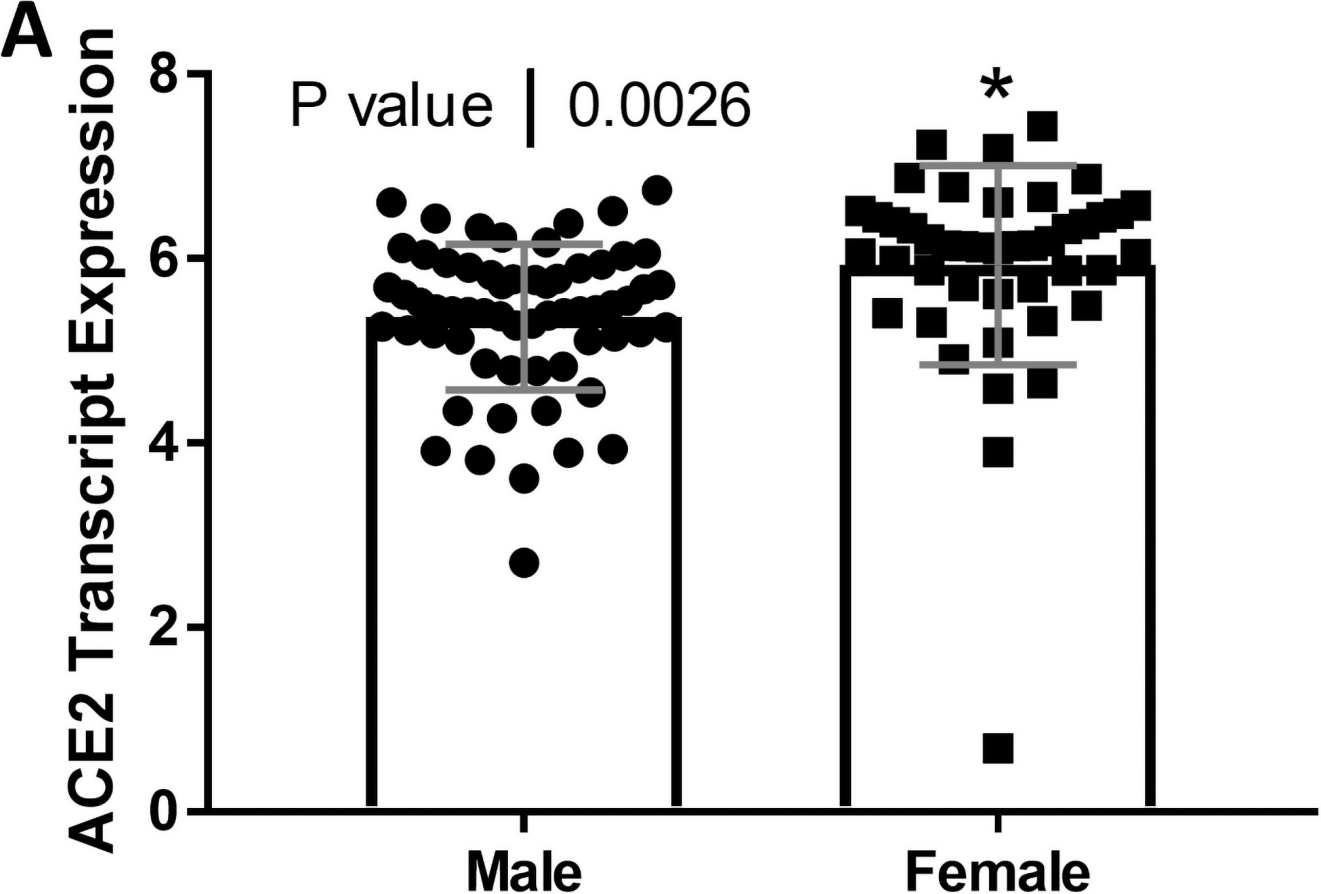
Tubular angiotensin-converting enzyme 2 (ACE2) mRNA expression in subjects with focal segmental glomerulosclerosis (FSGS). Tubular ACE2 mRNA expression (median-centered log2 expression by microarray analysis) in male and female subjects with FSGS. Values are the mean ± SD (grey lines). Significance was defined as a *P* value of less than 0.05.

**Fig 2:**
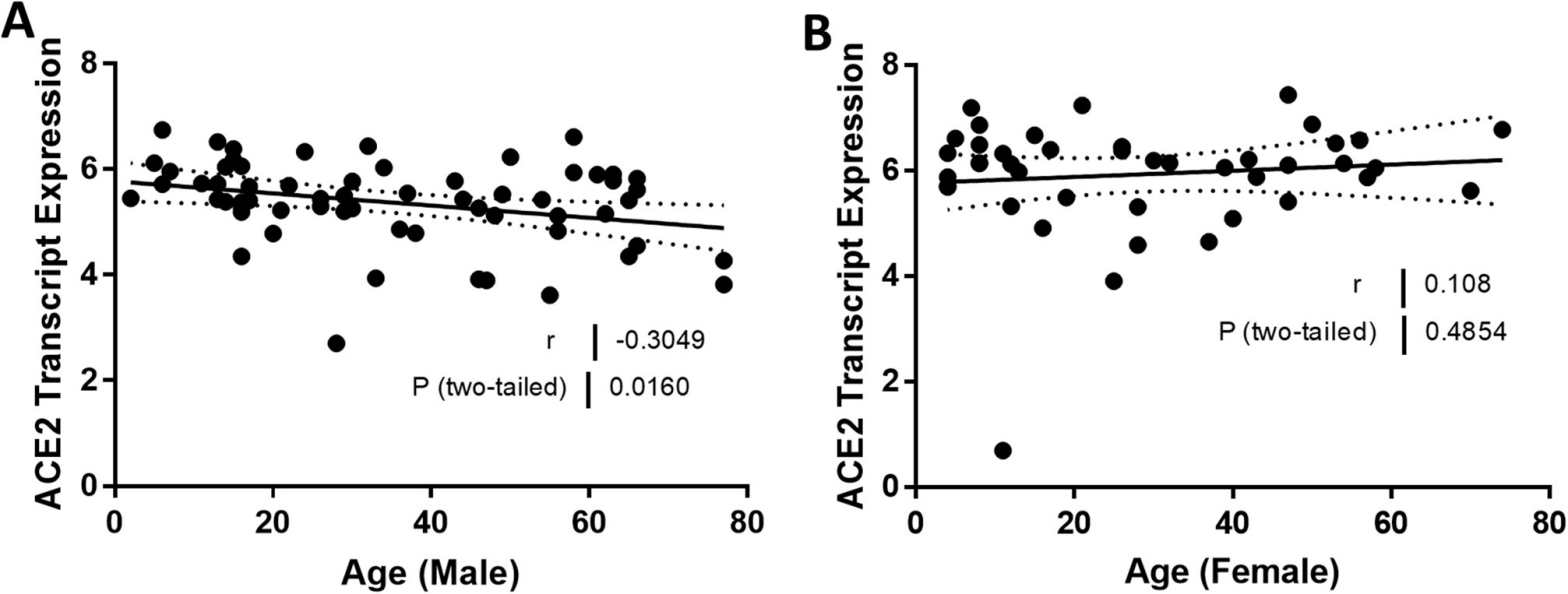
Correlation of tubular angiotensin-converting enzyme 2 (ACE2) mRNA expression with age in subjects with focal segmental glomerulosclerosis (FSGS). (A, B) Tubular ACE2 mRNA expression (median-centered log2 expression by microarray analysis) correlated with age in (A) male and (B) female subjects with FSGS. Pearson’s correlation coefficient (*r*) with two-tailed *P* values were calculated. Linear regression was used to generate the line of best fit (solid lines) with 95% confidence intervals (dotted lines). Significance was defined as a *P* value of less than 0.05.

**Fig 3:**
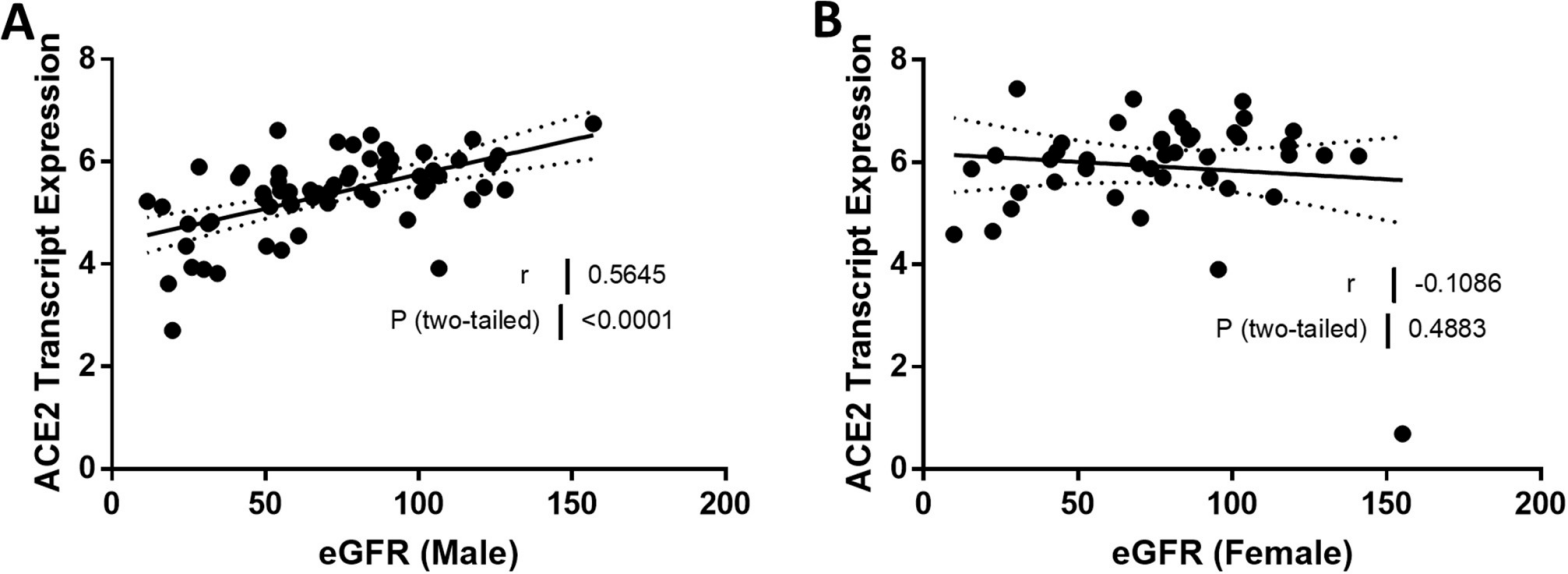
Correlation of tubular angiotensin-converting enzyme 2 (ACE2) mRNA expression with estimated glomerular filtration rate (eGFR) in subjects with focal segmental glomerulosclerosis (FSGS). (A, B) Tubular ACE2 mRNA expression (median-centered log2 expression by microarray analysis) correlated with eGFR in (A) male and (B) female subjects with FSGS. Pearson’s correlation coefficient (*r*) with two-tailed *P* values were calculated. Linear regression was used to generate the line of best fit (solid lines) with 95% confidence intervals (dotted lines). Significance was defined as a *P* value of less than 0.05.

**Fig 4:**
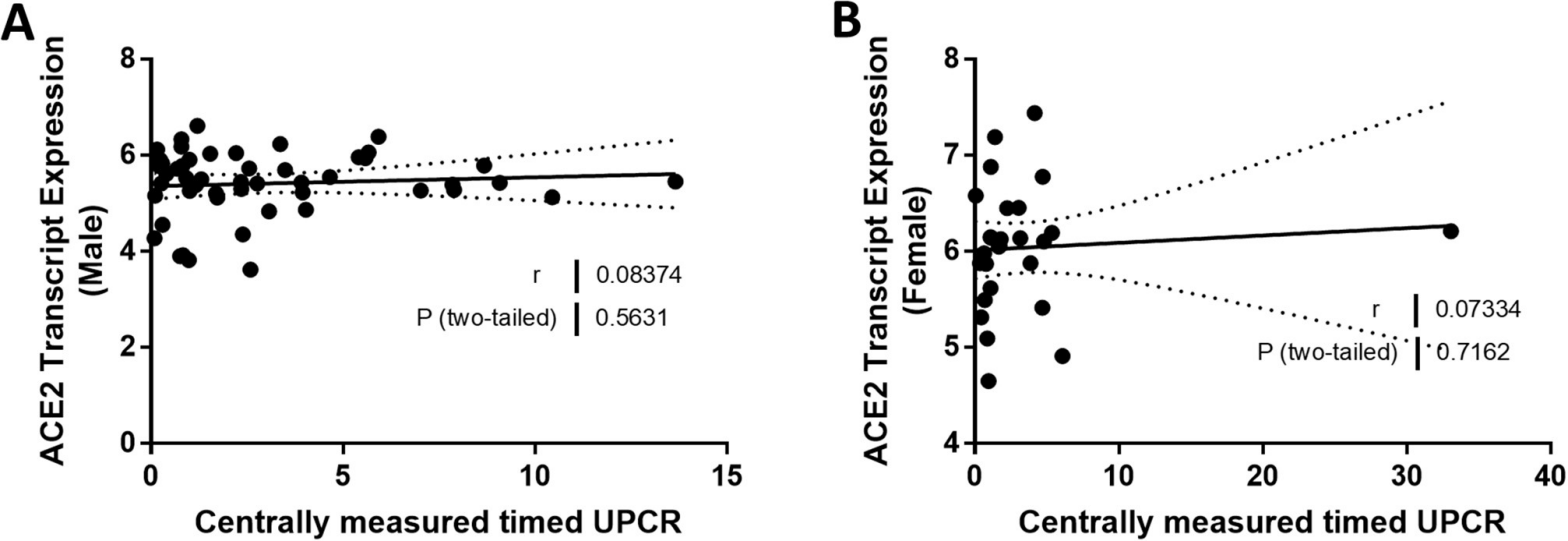
Correlation of tubular angiotensin-converting enzyme 2 (ACE2) mRNA expression with urine protein to creatine ratio (UPCR) in subjects with focal segmental glomerulosclerosis (FSGS). (A, B) Tubular ACE2 mRNA expression (median-centered log2 expression by microarray analysis) correlated with centrally measured timed UPCR in (A) male and (B) female subjects with FSGS. Pearson’s correlation coefficient (*r*) with two-tailed *P* values were calculated. Linear regression was used to generate the line of best fit (solid lines) with 95% confidence intervals (dotted lines). Significance was defined as a *P* value of less than 0.05.

**Fig 5:**
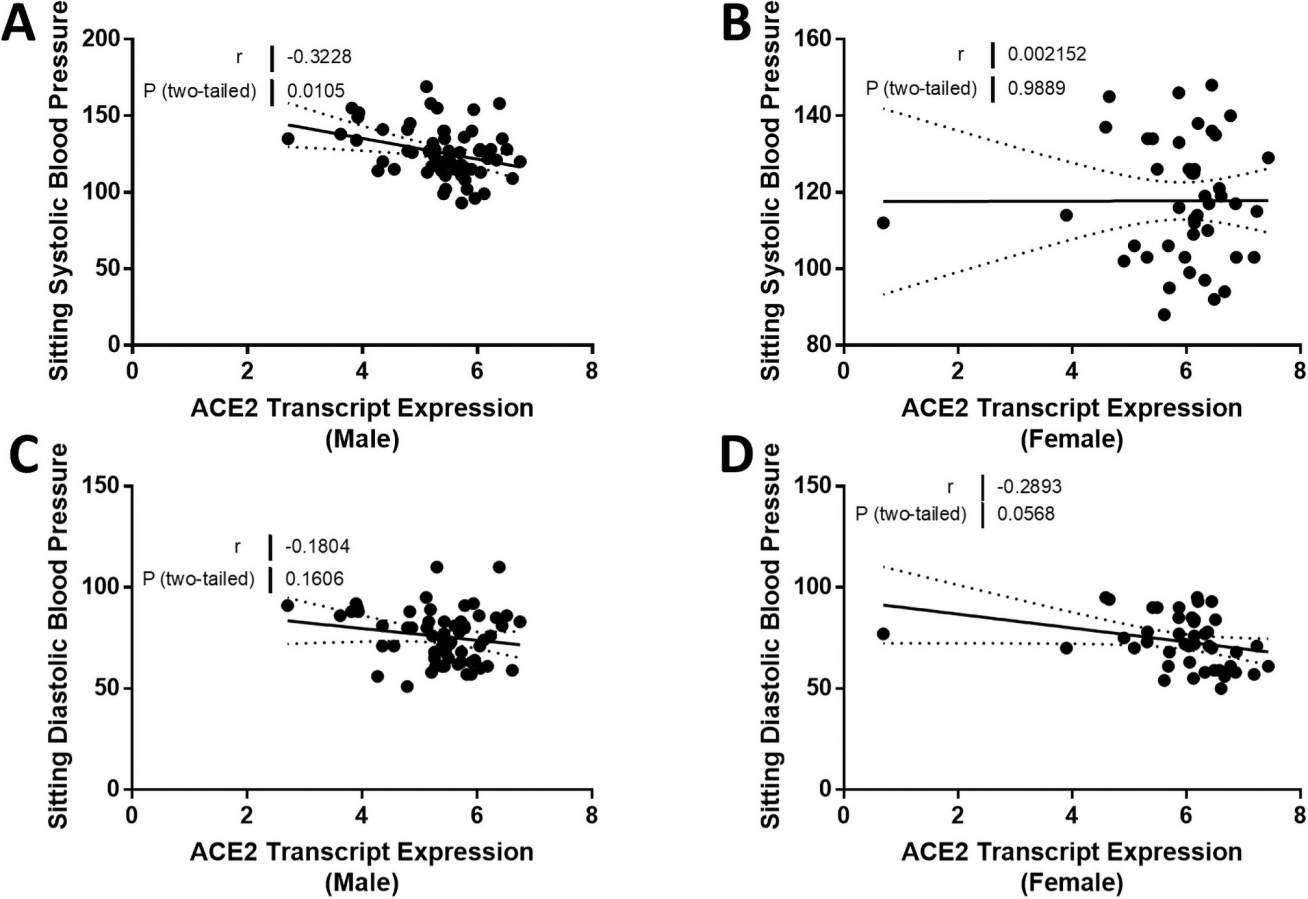
Correlation of tubular angiotensin-converting enzyme 2 (ACE2) mRNA expression with systolic and diastolic blood pressure in subjects with focal segmental glomerulosclerosis (FSGS). (A, B) Tubular ACE2 mRNA expression (median-centered log2 expression by microarray analysis) correlated with sitting systolic blood pressure in (A) male and (B) female subjects with FSGS. (C, D) Tubular ACE2 mRNA expression (median-centered log2 expression by microarray analysis) correlated with sitting diastolic blood pressure in (C) male and (D) female subjects with FSGS. Pearson’s correlation coefficient (*r*) with two-tailed *P* values were calculated. Linear regression was used to generate the line of best fit (solid lines) with 95% confidence intervals (dotted lines). Significance was defined as a *P* value of less than 0.05.

We compared ACE2 mRNA expression in the tubulointerstitium based on sex. Tubulointerstitial ACE2 mRNA expression was greater in females with FSGS compared to males with FSGS (*P* = 0.0026; Fig 1). In male subjects, ACE2 expression declined with age (*r* = -0.30, *P* = 0.016; Fig 2A), but this relationship was not observed in female subjects (*r* = 0.11, *P* = 0.50; Fig 2B). There was a relationship between ACE2 mRNA expression and eGFR in the tubulointerstitium in male subjects (*r* = 0.56, *P* < 0.0001; Fig 3A) but not in female subjects (*r* = -0.11, *P* = 0.49; Fig 3B). There were no relationships between the centrally measured timed UPCR values and ACE2 expression in either male (Fig 4A) or female subjects (Fig 4B). There was a relationship between sitting systolic blood pressure and ACE2 expression in males (r = -0.32, *P* = 0.010; Fig 5A).

**Fig 6:**
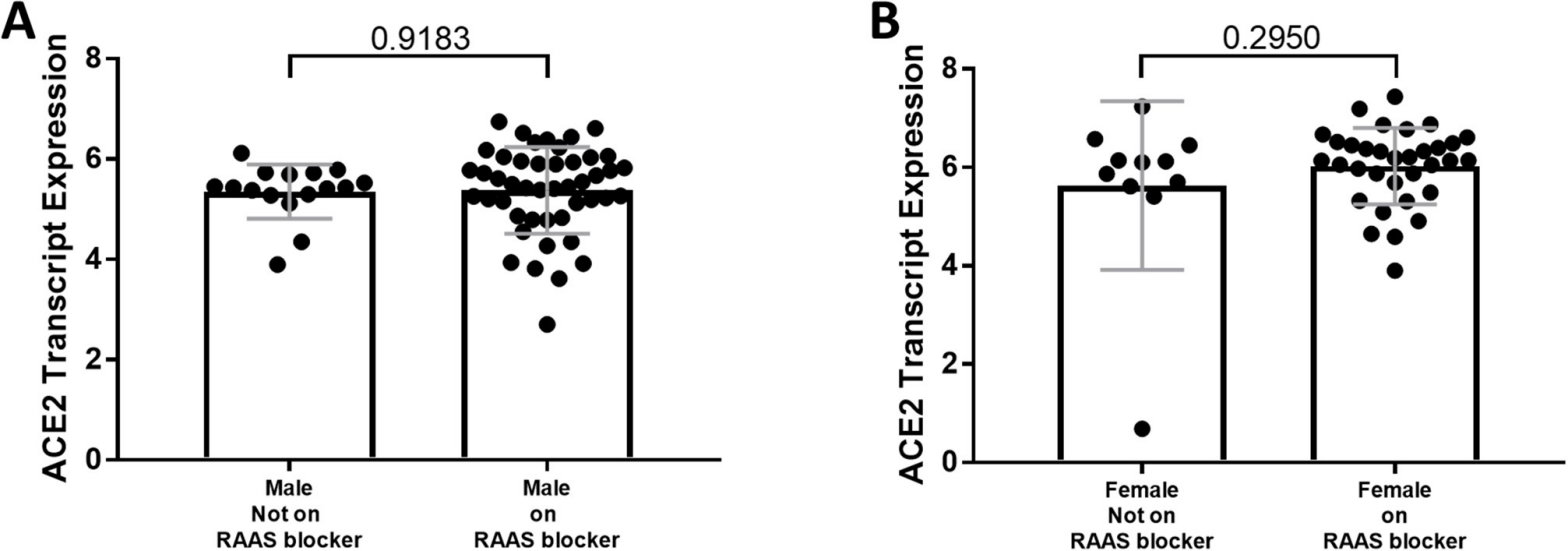
Comparison of tubular angiotensin-converting enzyme 2 (ACE2) mRNA expression in subjects with focal segmental glomerulosclerosis (FSGS) on or off RAAS blocker drugs. (A, B) Tubular ACE2 mRNA expression (median-centered log2 expression by microarray analysis) in (A) male and (B) female subjects with FSGS who were either on or not taking RAAS blockade drugs prior to the baseline visit. Values are the mean ± SD (grey lines). Significance was defined as a *P* value of less than 0.05.

A summary of the Spearman correlation coefficients and *P* values for ACE2 expression and clinical variables are shown in Table 3. The majority of males and females were treated with either angiotensin receptor blockers or angiotensin converting enzyme inhibitors at the time of kidney biopsy (Table 1). There was no effect of treatment on ACE2 expression in either male or female subjects (Fig 6). Multiple linear regression analysis showed that ACE2 expression was significantly related to sex and eGFR but not to age or treatment with renin angiotensin system blockade (Table 4). A one-unit increase in eGFR resulted in a 0.006 unit increase in ACE2 expression outcome. Women had on average 0.52 units higher ACE2 expression.

**Table 3:** Summary of correlation coefficients and *P* values for clinical variables that were correlated with ACE2 expression in male and female patients with FSGS.

	Male		Female	
	*r*	*P* value	*r*	*P* value
**Age**	-0.3049	0.0160	0.108	0.4854
**eGFR**	0.5645	< 0.0001	-0.1086	0.4883
**UPCR**	0.08374	0.5632	0.07334	0.7162
**Systolic BP**	-0.3228	0.0105	0.002152	0.9889
**Diastolic BP**	-0.1804	0.1606	-0.2893	0.0568
**IF**	-0.4654	0.0004	-0.098	0.4724
**TA**	-0.4601	0.0005	-0.04898	0.7734

BP, blood pressure; eGFR, estimated glomerular filtration rate; IF, interstitial fibrosis; TA, tubular atrophy; UPCR, urine protein to creatinine ratio.

**Table 4:** Multiple linear regression analysis.

Model: y = *β*_0_ + *β*_1_*x*_1_ + *β*_2_*x*_2_ + *β*_3_*x*_3_ + *β*_4_*x*_4_					
Predictor	Coefficient	Estimate	Standard Error	t-statistic	*p*-value
**Constant**	*β*_0_	4.8098	0.3709	12.967	0
**eGFR**	*β*_1_	0.0061	0.003	2.0591	0.0421
**Age**	*β*_2_	-0.0005	0.0049	-0.1048	0.9167
**Sex**	*Β*_3_	0.5197	0.1829	2.8411	0.0054
**RAASblock**	*Β*_4_	0.1887	0.2045	0.9226	0.3585

eGFR, estimated glomerular filtration rate; Age; Sex; RAASblock, renin-angiotensin-aldosterone system blocking drugs.

### Correlation of ACE2 mRNA expression with kidney interstitial fibrosis and tubular atrophy in males and females with FSGS

**Fig 7:**
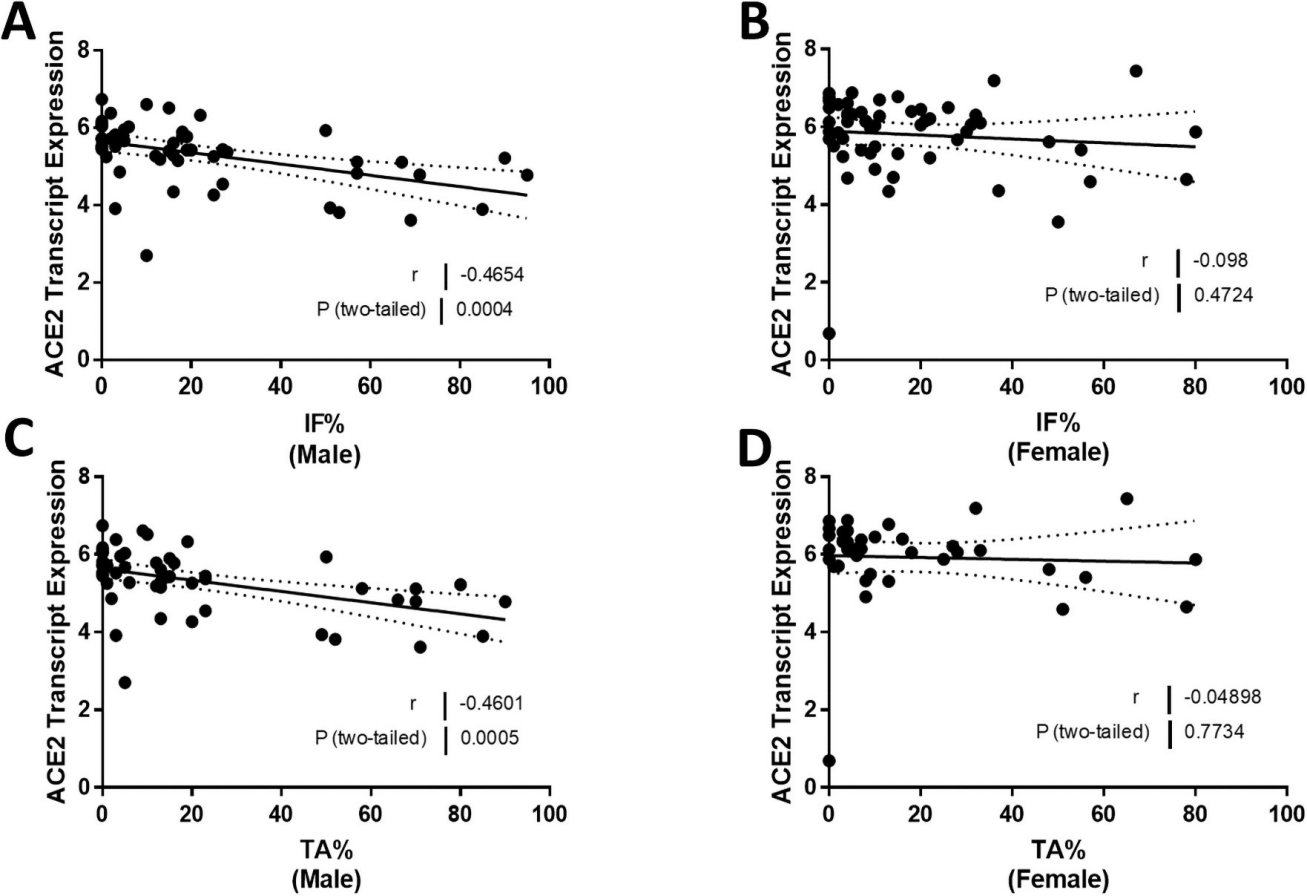
Correlation of tubular angiotensin-converting enzyme 2 (ACE2) mRNA expression with structural measurements in subjects with focal segmental glomerulosclerosis (FSGS). (A, B) Tubular ACE2 mRNA expression (median-centered log2 expression by microarray analysis) correlated with interstitial fibrosis percentage (IF %) in (A) male and (B) female subjects with FSGS. (C, D) Tubular ACE2 mRNA expression (median-centered log2 expression by microarray analysis) correlated with tubular atrophy percentage (TA %) in (C) male and (D) female subjects with FSGS. Pearson’s correlation coefficient (*r*) with two-tailed *P* values were calculated. Linear regression was used to generate the line of best fit (solid lines) with 95% confidence intervals (dotted lines). Significance was defined as a *P* value of less than 0.05.

We next compared the relationship between ACE2 expression and pathology measures of percent interstitial fibrosis and percent tubular atrophy in male and female subjects. In male subjects, ACE2 expression levels declined as values for percent interstitial fibrosis rose (*r* = -0.47, *P* = 0.0004; Fig 7A), but this relationship was not observed in female subjects (*r* = -0.10, *P* = 0.47; Fig 7B). There was also a relationship between ACE2 mRNA expression and percent tubular atrophy in male subjects (*r* = -0.46, *P* = 0.0005; Fig 7C) but not in female subjects (*r* = 0.05, *P* = 0.77; Fig 7D).

### Correlation of ACE2 mRNA expression with genes implicated in inflammation and fibrosis in males and females with FSGS

**Fig 8:**
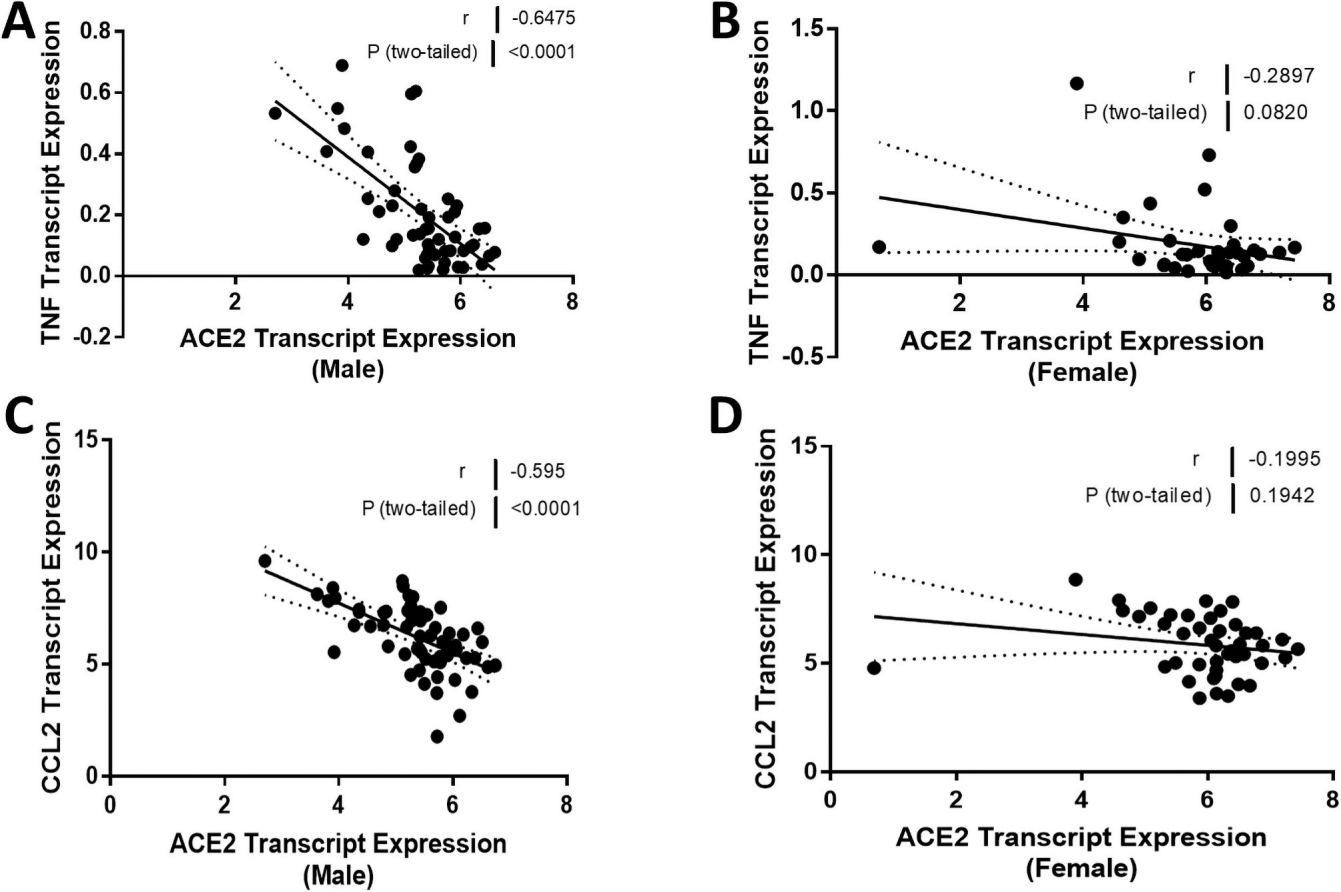
Correlation of tubular angiotensin-converting enzyme 2 (ACE2) mRNA expression with mRNA expression of inflammation genes in subjects with focal segmental glomerulosclerosis (FSGS). (A, B) Tubular ACE2 mRNA expression (median-centered log2 expression by microarray analysis) correlated with tumor necrosis factor (TNF) expression in (A) male and (B) female subjects with FSGS. (C, D) Tubular ACE2 mRNA expression (median-centered log2 expression by microarray analysis) correlated with monocyte chemoattractant protein 1 (MCP-1 or CCL2) expression in (C) male and (D) female subjects with FSGS. Pearson’s correlation coefficient (*r*) with two-tailed *P* values were calculated. Linear regression was used to generate the line of best fit (solid lines) with 95% confidence intervals (dotted lines). Significance was defined as a *P* value of less than 0.05.

**Fig 9:**
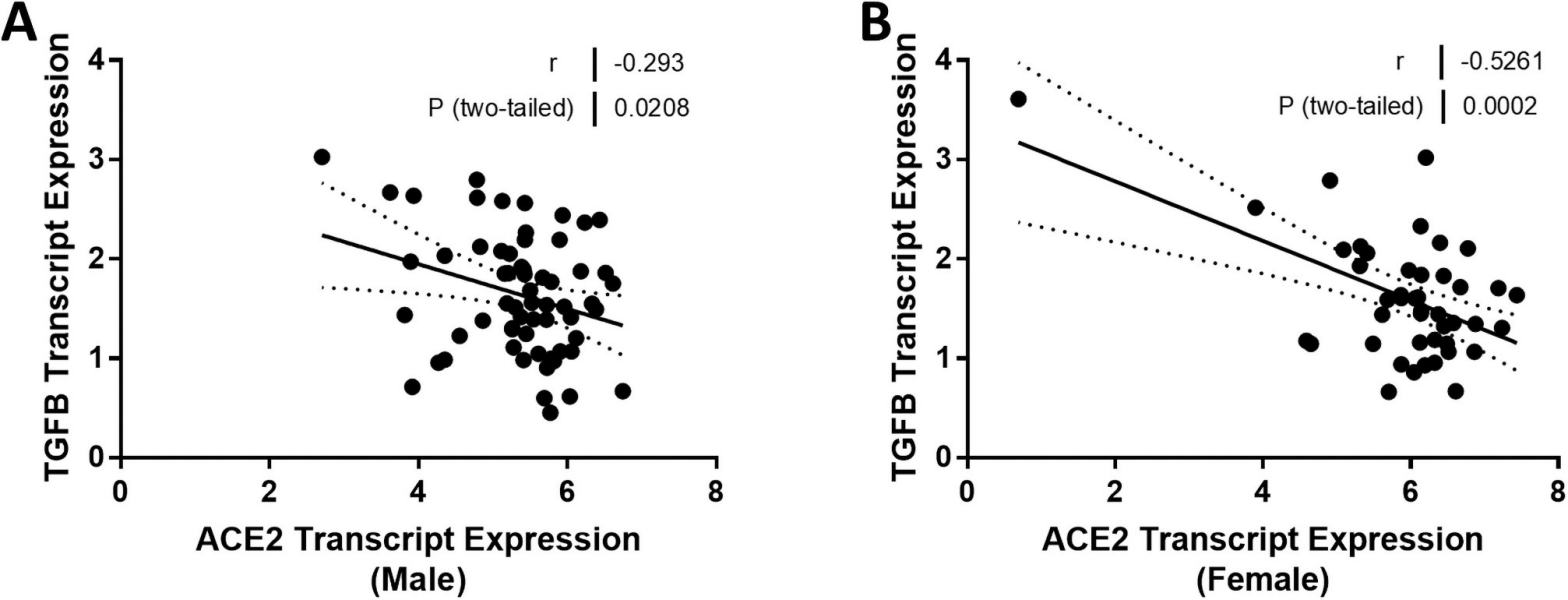
Correlation of tubular angiotensin-converting enzyme 2 (ACE2) mRNA expression with transforming growth factor beta (TGFB) mRNA expression in subjects with focal segmental glomerulosclerosis (FSGS). (A, B) Tubular ACE2 mRNA expression (median-centered log2 expression by microarray analysis) correlated with TGFB1 in (A) male and (B) female subjects with FSGS. Pearson’s correlation coefficient (*r*) with two-tailed *P* values were calculated. Linear regression was used to generate the line of best fit (solid lines) with 95% confidence intervals (dotted lines). Significance was defined as a *P* value of less than 0.05.

**Fig 10:**
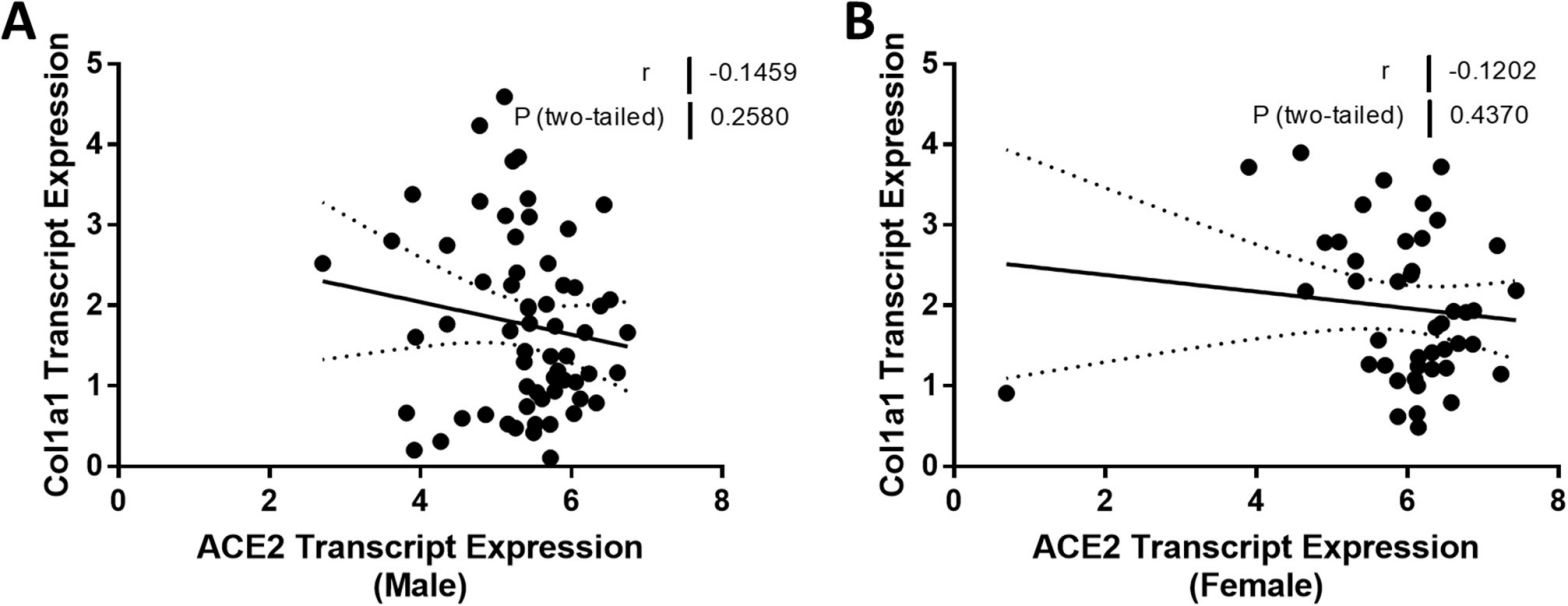
Correlation of tubular angiotensin-converting enzyme 2 (ACE2) mRNA expression with collagen, type I, alpha 1 (COL1A1) mRNA expression in subjects with focal segmental glomerulosclerosis (FSGS). (A, B) Tubular ACE2 mRNA expression (median-centered log2 expression by microarray analysis) correlated with COL1A1 in (A) male and (B) female subjects with FSGS. Pearson’s correlation coefficient (*r*) with two-tailed *P* values were calculated. Linear regression was used to generate the line of best fit (solid lines) with 95% confidence intervals (dotted lines). Significance was defined as a *P* value of less than 0.05.

**Fig 11:**
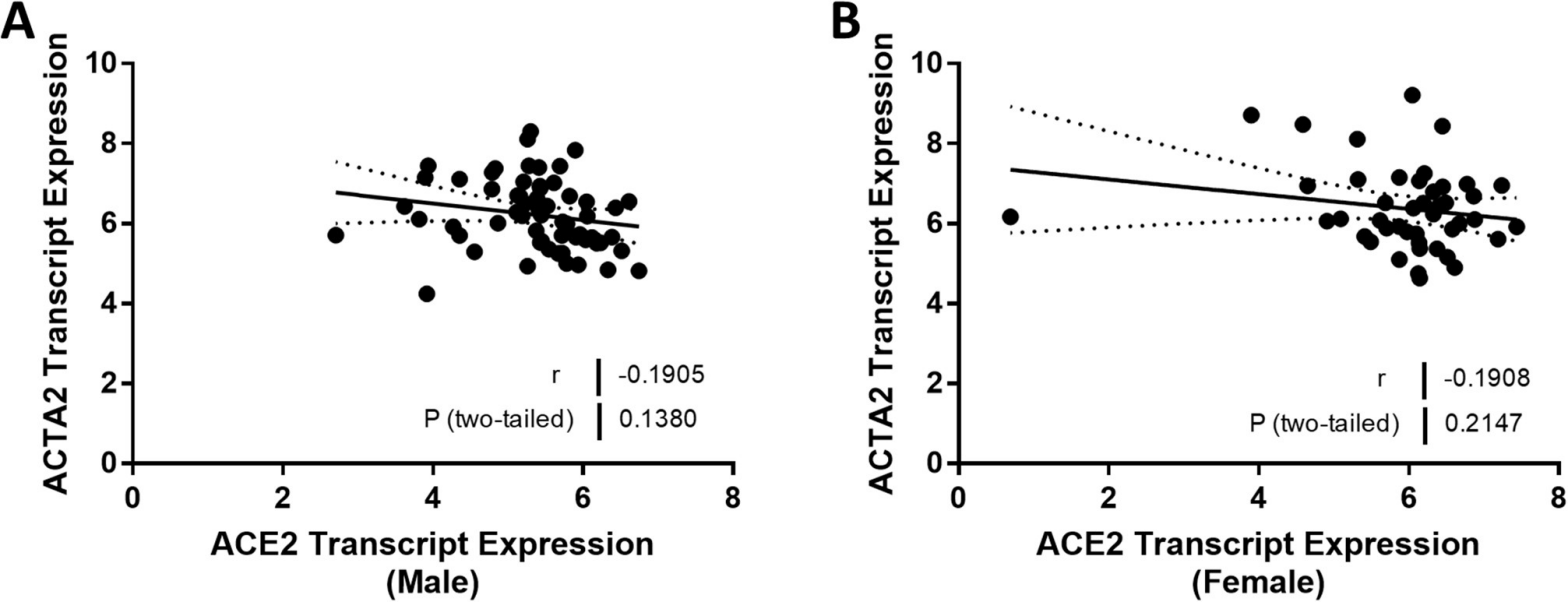
Correlation of tubular angiotensin-converting enzyme 2 (ACE2) mRNA expression with alpha smooth muscle actin (ACTA2) expression in subjects with focal segmental glomerulosclerosis (FSGS). (A, B) Tubular ACE2 mRNA expression (median-centered log2 expression by microarray analysis) correlated with ACTA2 in (A) male and (B) female subjects with FSGS. Pearson’s correlation coefficient (*r*) with two-tailed *P* values were calculated. Linear regression was used to generate the line of best fit (solid lines) with 95% confidence intervals (dotted lines). Significance was defined as a *P* value of less than 0.05.

Experimental studies have shown that ACE2 can regulate the expression of genes implicated in inflammation and fibrosis. Therefore, we compared the relationship between ACE2 expression and TNF and CCL2 mRNA in males and females. TNF mRNA levels were strongly associated with ACE2 mRNA levels (*r* = -0.65, *P* < 0.0001; Fig 8A) in men, but the relationship was not significant in females (*r* = -0.29, *P* = 0.08; Fig 8B). Our analysis showed a similar difference between males and females in the relationship between ACE2 expression and CCL2 expression (Fig 8C and 8D). We did not see any sexual dimorphism in the relationship between ACE2 expression and the expression levels of genes implicated in kidney fibrosis. TGFB was negatively associated with ACE2 expression in both males (*r* = -0.29, *P* = 0.02; Fig 9A) and females (*r* = -0.53, *P* = 0.0002; Fig 9B). There were no relationships between either ACE2 expression and COL1A1 or ACTA2 expression (Figs 10 and 11). A summary of the Spearman correlation coefficients and *P* values for ACE2 and the expression of genes implicated in inflammation and fibrosis is also shown in Table 5.

**Table 5:** Summary of correlation coefficients and *P* values for inflammation and fibrosis genes that were correlated with ACE2 expression in male and female patients with FSGS.

	Male		Female	
	*r*	*P* value	*r*	*P* value
**TNF**	-0.6475	< 0.0001	-0.2897	0.0820
**CCL2**	-0.595	< 0.0001	-0.1995	0.1942
**TGFB**	-0.293	0.0208	-0.5261	0.0002
**COL1A1**	-0.1459	0.2580	-0.1202	0.4370
**ACTA2**	-0.1905	0.1380	-0.1908	0.2147

ACTA2, actin alpha 2, smooth muscle; CCL2, monocyte chemoattractant protein 1; COL1A1, collagen, type I, alpha 1; TGFB, transforming growth factor beta; TNF, tumor necrosis factor.

## Discussion

The rationale for the current study is twofold. First, the RAS plays a key role in the pathogenesis of CKD associated with proteinuria, and blockade of the RAS limits CKD progression [27, 28]. The complexity of the RAS is evolving, and ACE2, an enzyme that processes Ang II, regulates Ang II-induced inflammation and fibrosis in the kidney [29]. Of current interest, ACE2 also functions as a cell membrane receptor for SARS-CoV and SARS-CoV-2. Notwithstanding these important functions, our knowledge of the determinants of ACE2 expression in the kidney is incomplete. Second, sex is an important determinant of kidney disease outcomes [18–20]. While the gene for ACE2 is on the X chromosome [30], studies of sex-based differences in kidney ACE2 expression and the relationships between ACE2 expression and kidney injury are limited. Accordingly, our goal was to study ACE2 expression in the NEPTUNE cohort [31] of FSGS and to compare relationships between ACE2 expression, clinical variables, and pathology in males and females.

We studied a well-characterized cohort of male and female subjects with FSGS [31]. We chose to study FSGS because clinical outcomes are dependent on sex: outcomes are better in females than males [23, 32]. For example, Troyanov and coworkers studied a cohort of patients with FSGS and found that female sex was associated with a sustained decrease in proteinuria, an important determinant of the rate of loss of kidney function [32]. In another study, kidney function declined more rapidly in men with FSGS than women [21]. Moreover, we have reported that there are sex differences in the kidney response to RAS blockade in females and males that may be due to differences in renal expression of components of the RAS [33]. Finally, we focused on the micro-dissected tubulointerstitial compartment of the kidney for two reasons. First, ACE2 is mainly in cells of the S1, S2, and S3 segments of the kidney proximal tubule [1, 2, 34], as shown by Wysocki and coworkers in a recent analysis of single cell transcriptomic data, and second, changes in the tubulointerstitium, in particular interstitial fibrosis and tubular atrophy, predict long term clinical outcomes in CKD [35, 36].

The FSGS cohort exhibited significant differences in systolic blood pressure, hematocrit values, and timed urine creatinine between males and females. The differences were expected based on population studies in females and males [37]. Our first observation was that ACE2 expression in the kidney tubulointerstitial compartment was lower in males than females (Fig 1). Interestingly, ACE2 is on the X chromosome [30]. Although the presence of two X chromosomes (and therefore two ACE2 genes) might cause a two-fold increase in ACE2 expression in females relative to males, X chromosome inactivation will lessen this difference. Genes on one of the X chromosomes from either parent are marked for inactivation. This leads to the silencing of genes on that chromosome leading to a gene dosage more comparable to males. In reality, incomplete inactivation occurs in human female tissues. Genes that escape inactivation may have higher levels of expression, but likely less than a two-fold increase when compared to males [38]. Tukiainen and coworkers profiled “escape genes” in multiple tissues in humans [39]. Interestingly they found that ACE2 expression was higher in males for most of the tissues profiled. However, they did not study kidney expression. These findings suggest that we should not expect to see a large difference in kidney ACE2 expression based on sex chromosomes alone. Alternatively, sex hormones (testosterone and 17β-estradiol) may regulate the expression of ACE2 [39, 40]. For example testosterone may upregulate ACE2 expression, and reduced testosterone levels with aging might account, at least in part, for the relationship between ACE2 expression and age in males [41, 42]. In addition, estradiol may also impact ACE2 activity in the kidney [43]. We also found that ACE2 expression was lower in men compared to women in a recent study in a cohort of subjects with chronic kidney disease due to a variety of pathologies [23]. ACE2 expression in the kidney declined in age but only in men [23].

Our next observation was that there was a relationship between ACE2 expression and eGFR in males but not in females, even though the distribution of eGFR values was similar in both groups (Fig 3). As values for eGFR decline, there is a decrease in ACE2 expression. Taken together, our findings of dichotomies in the relationships between ACE2 expression and sex, age, and eGFR in males and females suggests that male kidneys may be more vulnerable to kidney disease associated with activation of the RAS. Ang II drives both inflammation and fibrosis in the kidney [10, 44]. ACE2 is responsible for the proteolytic degradation of Ang II to Ang-(1–7), and reduced ACE2 expression ought to lead to an increase in Ang II levels in the kidney [45]. Infusing Ang II into mice with a deletion in the gene for ACE2 is associated with more fibrosis and inflammation compared to infusion in a wildtype mouse [46]. The RAS is activated in kidney disease associated with proteinuria [47]. Indeed, Remuzzi and coworkers reported that the clinical benefit of RAS blockade was dependent on the level of proteinuria [48]. More recently, Matsusaka and coworkers showed that liver-derived angiotensinogen contributed to increased kidney Ang II levels when glomerular injury was associated with impaired permselectivity [49]. Any reduction in ACE2 expression should lead to further increases in tissue Ang II levels Sex and age differences in ACE2 expression may well contribute, at least in part, to clinical outcomes in FSGS.

RAAS blockade may also affect tissue ACE2 expression [50], Danser and coworkers recently reported that neither angiotensin converting enzyme inhibition (ACEI) nor angiotensin receptor blockers (ARB) consistently increased lung and kidney ACE2 expression, although the focus of this work was on potential susceptibility to coronavirus infection and the role of ACE2 as a receptor for cellular viral entry [51]. In addition, Cahoya and coworkers reported that RAS blockade did not change ACE2 expression in kidney allografts, albeit by immunohistochemistry in a small cohort of subjects [52]. In contrast, Wysocki and coworkers found that RAS treatment in mice decreased kidney ACE2 protein expression [53]. We therefore compared ACE2 mRNA expression in female and male subjects treated with RAAS blockade in our cohort of FSGS subjects. ACE2 expression was similar in untreated and treated males and females suggesting that RAAS blockade did not have a major effect on kidney ACE2 expression in the cohort.

Given the above findings, we studied the impact of eGFR, age, sex, and the use of RAAS blockade on ACE2 expression in our cohort, we performed a multiple linear regression analysis in which we related ACE2 expression to eGFR, age, sex, and the use of renin-angiotensin-aldosterone system blocking drugs prior to time of biopsy. Neither age nor RAAS blockade were associated with ACE2 expression outcome in the multiple linear regression analysis.

In our previous report, we were unable to relate ACE2 expression in the kidney to kidney fibrosis [23]. Accordingly, our next observation was the discovery that there was a significant relationship between ACE2 expression in the kidney and both interstitial fibrosis and tubular atrophy, but only in males and not in females (Fig 7). As ACE2 expression falls, interstitial fibrosis and tubular atrophy measures rise in males, in accord with the expected effect on Ang II levels. These relationships do not establish causality, although we have reported that treatment with recombinant ACE2 limits kidney fibrosis in an experimental murine model of Alport syndrome that is also associated with the development of FSGS [10]. Interestingly, 17β-estradiol increases ACE2 expression [54], and this may account for the failure to observe any relationship between ACE2 expression and interstitial fibrosis and tubular atrophy in the kidney of females with FSGS. Interestingly, there was a negative correlation between ACE2 expression and TGFB expression in both men and women, whereas there were no relationships, only trends, between ACE2 expression and COL1A1 and ACTA2 in both sexes.

Our next observations were the associations between ACE2 expression and gene expression of cytokines related to inflammation (Fig 8). There were strong negative correlations between ACE2 expression and TNF and CCL2 expression in males. Similar trends were observed in females although the relationships did not achieve statistical significance for either cytokine. The sexual dichotomy was marked by differences in the strength of the correlation, although this may be due in part to the number of data points. Taken together, the findings support the hypothesis that reduced ACE2 expression may contribute to increased kidney inflammation. This assertion is supported again by our observations of Ang II infusion in mice with a deletion in the gene for ACE2 [46] and by our experimental findings of ACE2 treatment in murine Alport syndrome [10, 27].

Studies of ACE2 are timely, especially in the context of its role as a cellular receptor for SARS-CoV-2 that facilitates viral cell entry [55]. CKD and male sex are risk factors for both SARS-CoV-2 infection and COVID-19 disease severity [42, 56–58]. Although acute kidney injury is a common complication of severe COVID-19 [58], the role of direct viral infection of the kidney is not well understood [59, 60]. Much of the acute risk relates to a diffuse systemic inflammatory response and renal hypoperfusion. We did not study acute SARS-CoV-2 infection in humans with FSGS. The sex and age-dependent changes in kidney ACE2 expression that we found do not appear to account for the impact of these clinical factors on COVID-19 infection and disease severity, although there may be tissue-specific relationships that are important in other organs [61]. Nevertheless, we would argue that tissue depletion of ACE2 in the setting of viral infection is likely to promote inflammation and scarring, and this may be particularly important in both acute lung infection and chronic lung injury [62].

Our study has several strengths. The cohort is well characterized in terms of clinical variables, such as assessment of blood pressure, eGFR, proteinuria, and measures of IF and TA in kidney biopsy samples [31, 63]. This allowed us to study the relationships between several variables and ACE2 expression, perhaps most importantly, histological assessment of kidney fibrosis.

However, our study also has some important limitations. First, our analysis is limited to mRNA levels in micro-dissected kidney tubulointerstitial samples, and we did not perform *in situ* hybridization studies of biopsy tissue from our cohort. The localization of ACE2 expression is important, and in this regard, a recent analysis of publicly available single cell transcriptomic data by Wysocki and co-workers showed that ACE2 is mainly in kidney proximal tubule S1, S2, and S3 cells [53]. We have extended this analysis by performing an *in-silico* analysis of two publicly available single cell transcriptomic databases (S1 Fig) to show proximal tubule cell-specific localization of ACE2 in both adult and fetal kidney tissue. Although it is important to note that glomerular cells are relatively under-represented in these datasets, measures of mRNA levels in the micro-dissected tubulointerstitial datasets we analyzed likely reflect changes in the proximal tubule. We did not relate ACE2 mRNA levels to ACE2 protein expression by immunohistochemistry in our cohort. Studies have reported tissue ACE2 protein expression by immunohistochemistry in humans [13] and confirmed the localization to proximal tubule cells [13], but there are only a few case series of ACE2 immunohistochemistry in human kidney biopsy samples [2, 14–17].

Another major limitation of our work is that we did not relate kidney ACE2 mRNA to levels of plasma Ang II and Ang-(1–7). Measures of intra-renal RAS activity and systemic RAS activity may not always be concordant, as noted in studies by Brenner and coworkers [64]. We have also found inconsistencies between systemic and renal measures of the RAAS in our recent studies of youth with type 2 diabetes that limit interpretation [65]. Serfero et al have suggested that the production of Ang-(1–7) from Ang II in the circulation is independent of ACE2 [66]. In contrast to this observation, we have reported that kidney Ang II and Ang-(1–7) levels are dependent, at least in part, on renal ACE2 [10]. Taken together, these findings suggest that systemic (circulating) measures of circulating Ang II and Ang-(1–7) may not reliably reflect intra-renal levels. Nevertheless, measures of kidney Ang II and Ang-(1–7) would strengthen our work and should be the focus of future studies.

In conclusion, kidney ACE2 expression in the tubulointerstitium differs in males and females with FSGS. ACE2 expression is greater in females than in males. Multiple linear regression analysis confirmed that the effect of sex was independent of eGFR, age, and blockade of the RAAS. The associations between ACE2 expression and eGFR, interstitial fibrosis, and tubular atrophy in males may account, at least in part, for the differences in clinical outcomes between males and females with FSGS. Finally, lower ACE2 expression in males is associated with the higher expression of genes implicated in inflammation.

## Supporting information

S1 Fig Transcriptomic analysis of ACE2 expression. Cell Clustering and ACE2 expression from the Kidney Interactive Transcriptomics (KIT), healthy human adult kidney (http://humphreyslab.com/SingleCell) and expression atlas, human fetal kidney (https://www.ebi.ac.uk/gxa/home).
(TIF)

S1 Dataset Clinical characteristics, RAAS patient drug information, and log2 median-centered microdissected tubulointerstitial biopsy microarray data. This file includes all the data that was used for analysis in this manuscript.
(XLSX)
