# Supplementary figures and images for: Sex and kidney ACE2 expression in primary focal segmental glomerulosclerosis: A NEPTUNE study
Source: PLoS One. 2021 Jun 7;16(6):e0252758. doi: 10.1371/journal.pone.0252758 (PMC8184004; doi:10.1371/journal.pone.0252758)

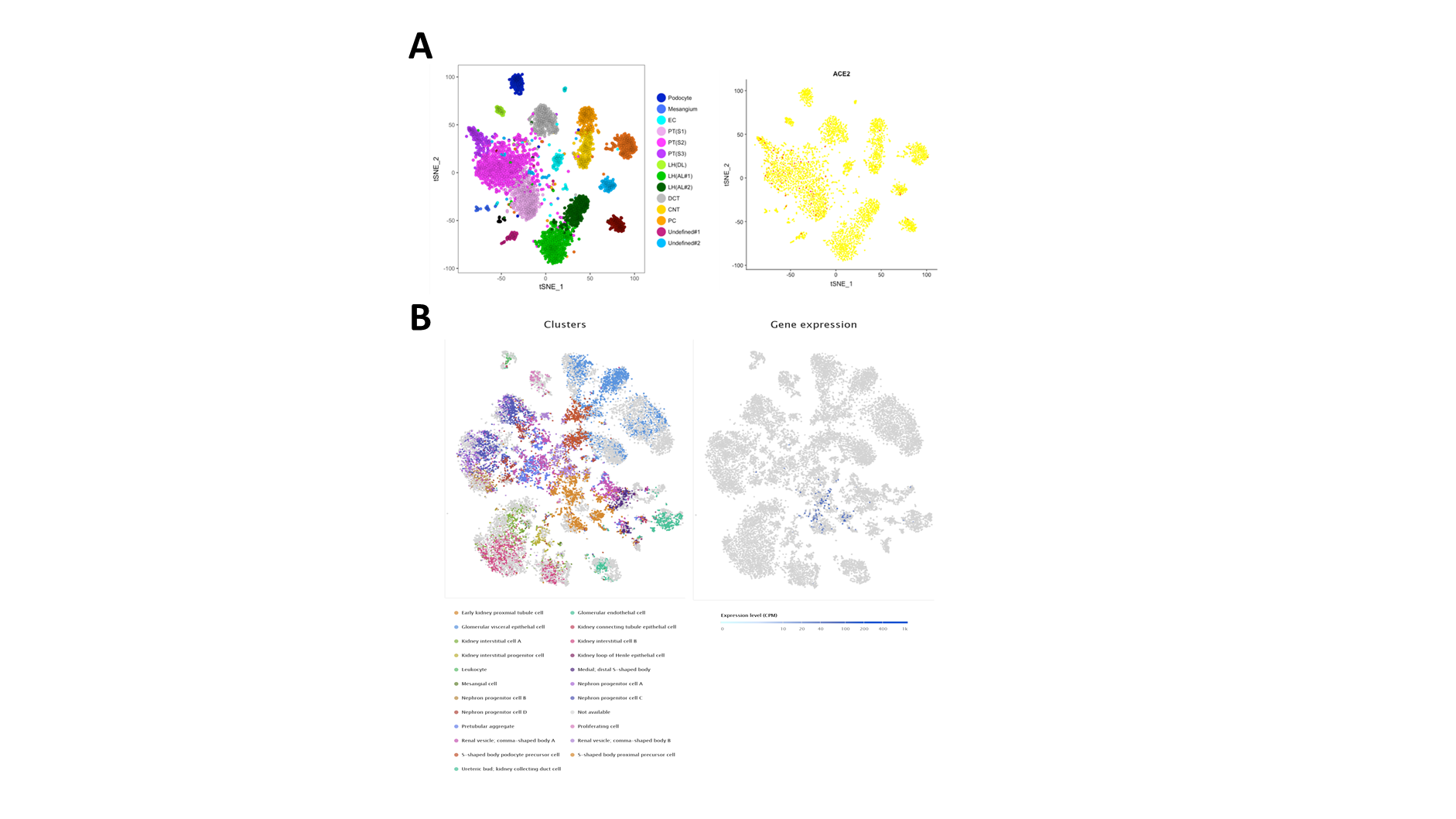

Supplement: S1 Fig — Cell Clustering and ACE2 expression from the Kidney Interactive Transcriptomics (KIT), healthy human adult kidney (http://humphreyslab.com/SingleCell) and expression atlas, human fetal kidney (https://www.ebi.ac.uk/gxa/home). (TIF) [file pone.0252758.s001.tif]
